# Identification of Proteins Enriched in Rice Egg or Sperm Cells by Single-Cell Proteomics

**DOI:** 10.1371/journal.pone.0069578

**Published:** 2013-07-25

**Authors:** Mafumi Abiko, Kensyo Furuta, Yoshio Yamauchi, Chiharu Fujita, Masato Taoka, Toshiaki Isobe, Takashi Okamoto

**Affiliations:** 1 Department of Biological Sciences, Tokyo Metropolitan University, Tokyo, Japan,; 2 Department of Chemistry, Tokyo Metropolitan University, Tokyo, Japan; University of Hawaii at Manoa, John A. Burns School of Medicine, United States of America

## Abstract

In angiosperms, female gamete differentiation, fertilization, and subsequent zygotic development occur in embryo sacs deeply embedded in the ovaries. Despite their importance in plant reproduction and development, how the egg cell is specialized, fuses with the sperm cell, and converts into an active zygote for early embryogenesis remains unclear. This lack of knowledge is partly attributable to the difficulty of direct analyses of gametes in angiosperms. In the present study, proteins from egg and sperm cells obtained from rice flowers were separated by one-dimensional polyacrylamide gel electrophoresis and globally identified by highly sensitive liquid chromatography coupled with tandem mass spectroscopy. Proteome analyses were also conducted for seedlings, callus, and pollen grains to compare their protein expression profiles to those of gametes. The proteomics data have been deposited to the ProteomeXchange with identifier PXD000265. A total of 2,138 and 2,179 expressed proteins were detected in egg and sperm cells, respectively, and 102 and 77 proteins were identified as preferentially expressed in egg and sperm cells, respectively. Moreover, several rice or *Arabidopsis* lines with mutations in genes encoding the putative gamete-enriched proteins showed clear phenotypic defects in seed set or seed development. These results suggested that the proteomic data presented in this study are foundational information toward understanding the mechanisms of reproduction and early development in angiosperms.

## Introduction

In angiosperms, the female gametophyte, referred to as the embryo sac, develops from a functional megaspore via three or more rounds of mitosis without cytokinesis. Subsequently, plasma-membranes/cell-walls are formed between the nuclei, resulting in a cellularized female gametophyte, generally known as an embryo sac. The embryo sac most commonly consists of one egg cell, one central cell, two synergid cells, and three antipodal cells [1], [2] and plays critical roles in pollen tube guidance, double fertilization, and seed development [3], [4]. In the anthers, male microspores undergo an initial asymmetric mitotic division, and the smaller generative cell, which establishes the male germ line, migrates into larger vegetative cell. The generative cell within the vegetative cell divides into two sperm cells before or after germination of the pollen tube.

Upon double fertilization, one sperm cell from the pollen tube fuses with the egg cell, and the resultant zygote develops into an embryo transmitting genetic material from the parents to the next generation. The central cell fuses with the second sperm cell to form a triploid primary endosperm cell, which develops into the endosperm nourishing the developing embryo and later seedling [5]–[7]. Within the embryo sac, the haploid egg cell is specially differentiated for fertilization and subsequent embryogenesis. However, it remains unclear how the egg cell specializes, fuses with the sperm cell, and converts into an active zygote for early embryogenesis, despite its importance in plant reproduction and development. This lack of knowledge is partly attributable to the fact that gametogenesis, fertilization, and embryogenesis all occur in the embryo sac, which is deeply embedded within the ovary, making direct observation and characterization of the female and male gametes in the embryo sac difficult.

Recently, single-cell proteomic approaches have been widely employed to dissect the functions of specific cells, because cellular-level information is diluted when organs or tissues, which comprise various differentiated cells, are used as starting materials [8]. For example, more than 1,000 unique proteins have been identified in pollen grains [9], guard cells [10]–[12], trichomes [13], [14], and root hairs [15], [16]. However, such global proteome analyses have not been conducted for plant egg and sperm cells, presumably because of the difficulty in obtaining sufficient, highly-pure homogenous cells, especially for egg cells. Procedures for isolating viable gametes have been reported for a wide range of plant species, including maize, wheat, tobacco, rape, rice, barley, *Plumbago zeylanica,* and *Alstroemeria* [17]–[19]. We previously conducted proteomic analyses using 75–290 maize and rice egg cells, although only six and four major protein components could be identified, respectively [20], [21]. However, in those studies, trace amount of proteins in just 10–20 egg cells could be detected by minimizing the size of gels. Moreover, state-of-the-art proteomics technologies enable high throughput and high-resolution analyses using such limited numbers of cells.

In the present study, large numbers of rice gametes were isolated from flowers, and proteins expressed in egg and sperm cells were globally detected by highly sensitive liquid chromatography coupled with tandem mass spectroscopy (LC-MS/MS) technology, and proteins which are preferentially expressing in gametes were identified by comparison of protein expression profiles between gametes and somatic cells/pollen grains. In addition, it is supposed that the gamete-enriched proteins function in reproductive and/or developmental processes such as gamete differentiation, gamete fusion, early zygotic development, and that defects in function of these proteins affect such biological processes. Therefore, seed-set fertility of the rice plant possessing transposon-insertional mutation for the several genes encoding the putative gamete-enriched proteins was checked. Moreover, the *Arabidopsis* plants possessing T-DNA-insertional mutations for putative orthologous genes to rice gene encoding the putative gamete-enriched protein were obtained, and profiles of seed development of these mutants were observed. Several of these rice and *Arabidopsis* mutants showed clear phenotypes of fertility defects, suggesting that the present proteomic results for rice gametes are useful basic information for understanding the reproductive and/or developmental processes in angiosperms.

## Materials and Methods

### Plant Materials

*Oryza sativa* L. cv. Nipponbare was grown in environmental chambers (K30-7248, Koito Industries, Yokohama, Japan) at 26°C in a 13/11 h light/dark cycle, and gametes were isolated from flowers. Tos17 insertional rice plants were obtained from Rice Genome Resource Center, National Institute of Agrobiological Sciences (NIAS, Tsukuba, Japan), and grown in an experimental field at our university from mid-May to October to check fertility. *Arabidopsis thaliana* ecotype Columbia and T-DNA insertion lines obtained from the Arabidopsis Biological Resource Center (ABRC, Columbus, OH, USA) were grown in an air-conditioned room at 23°C in a 16/8 h light/dark cycle.

### Isolation of Gametes for Proteomic Analyses

Rice egg cells were isolated according to Uchiumi et al. [22], except that the mannitol solution was adjusted to 370 mOsmol/kg H_2_O instead of 0.3 M mannitol (Fig. S1). After washing the cells three times by transferring the cells into fresh droplets of mannitol solution on coverslips, fifty to seventy isolated egg cells were transferred into a 1 µL droplet of SDS-sample buffer (2% SDS, 25 mM Tris-Cl pH 6.8, 30% glycerol, 5% 2-mercaptoethanol) and treated at 98°C for 3 min, then stored at –80°C until use.

Sperm cells were isolated according to Zhang et al. [23] and Gou et al. [24] with modifications. Approximate 120 anthers harvested from rice flowers were collected in plastic dishes (φ 3.5 cm) filled with 3 mL of 370 mOsmol kg^−1^ H_2_O mannitol. After washing the anthers by gentle shaking, the anthers were transferred to four plastic dishes (φ 3.5 cm) filled with 3 mL of 15% sucrose, and the tissues were broken with forceps to free the pollen grains. After gentle shaking for 30 min, the sucrose solution, in which pollen grains released their sperm cells, was filtered twice, through 20-µm then 10-µm nylon bolting cloth. To the filtrate, an equal volume of 15% sucrose containing 60% Percoll (GE Healthcare UK Ltd., UK) was added, and then 4 mL of the mixture was transferred into a 13PA centrifugation tube (Hitachi, Japan). Over the mixture, 20% and 5% Percoll in 15% sucrose were layered to form a discontinuous Percoll gradient in the tube. After centrifugation at 3,000 ×*g* for 30 min at 4°C, the interface between the 5% and 20% Percoll layers was collected, and an equal volume of 15% sucrose was added to the collected fraction. Then, the fraction, containing sperm cells, was centrifuged at 5,000 ×*g* for 4 min at 4°C. The bottom 15 µL of the tube, containing concentrated sperm cells, was used as the isolated sperm cells after counting the number of cells in the fraction. An equal volume of SDS-sample buffer was added to the fraction, and the mixture was treated 98°C for 3 min then stored at –80°C until use.

### Preparation of Lysates from Rice Callus, Seedlings, and Pollen Grains

Pollen grains released from 10–20 anthers were homogenized with 0.5 mL of SDS-sample buffer using a mortar and pestle. Rice seeds were cultured on N6D medium containing 2,4-D for 7 days at 30°C under the continuous light according to Toki et al. [25], and the callus derived from scutellum were harvested. For obtaining seedlings, rice seeds were sown in water and grown at 26°C in darkness for 4 days, and then the germinated seeds were further grown at 26°C in a 13/11 h light/dark cycle for 7 days. The seedlings whose shoot length are 2.5–3 cm were harvested. Callus (0.3 g) or five seedlings were homogenized with 0.4 mL of SDS-sample buffer using a mortar and pestle. Each homogenate was treated at 98°C for 5 min, then centrifuged for 10,000×*g* for 5 min at room temperature. The supernatants were used as lysates from pollen grains, callus, and seedlings, respectively, and stored at –80°C until use. Lysate protein concentrations were measured using the Pierce 660 nm protein assay kit (Thermo Scientific, MA, USA) using bovine plasma gamma globulin (Bio Rad, CA, USA) as standard.

### SDS-polyacrylamide Gel Electrophoresis

According to Laemmlie [26], 12.5% SDS-polyacrylamide (SDS-PAGE) gels were prepared in a small mold (50×60×1 mm; Atto, Tokyo, Japan), and cell lysates from egg cells, sperm cells, seedlings, callus, and pollen grains in SDS-sample buffer were separated. Proteins in the gel were detected by conventional silver staining [27]. When gels were used for subsequent LC-MS/MS analyses, proteins in the gel were visualized with modified silver staining according to Taoka et al. [28].

### Identification of Proteins by Tandem Mass Spectrometry

SDS-PAGE gels were cut into 15 pieces. Proteins in each piece were in-gel-digested with trypsin [29] and identified by liquid chromatography coupled with tandem mass spectroscopy (LC-MS/MS) using a direct nanoflow LC-MS system equipped with an Orbi Trap XL (Thermo Scientific) mass spectrometer as described elsewhere [30]. Dataset of protein sequences obtained from the Rice Annotation Project Database (Tsukuba, Japan; http://rapdb.dna.affrc.go.jp/download/irgsp1.html) was searched using Mascot software (ver. 2.2.1, Matrix Science, MA, USA) with the following parameters. The fixed modification was propionasmide (Cys) and variable modification parameters were pyro-Glu, acetylation (protein N-terminus), and oxidation (Met). The maximum missed cleavage was set at 3 with a peptide mass tolerance of +/– 15 ppm. Peptide charges from +2 to +4 states and MS/MS tolerances of +/– 0.8 Da were allowed. The criteria for peptide identification were based on the vendor’s definitions (expectation value < 0.05, Matrix Science), and we assigned the protein “identified” if at least two peptides were identified from the protein. The mass spectrometry proteomics data have been deposited to the ProteomeXchange Consortium (http://proteomecentral.proteomexchange.org) via the PRIDE partner repository [31] with the dataset identifier PXD000265 and DOI 10.6019/PXD000265.

### cDNA Synthesis and Quantitative PCR

Rice egg cells were isolated as above, and sperm cells were manually isolated according to Uchiumi et al. [22]. Isolated egg or sperm cells were washed three times by transferring the cells into fresh droplets of mannitol solution on coverslips. The washed cells were submerged in 5 µl of the extraction buffer supplied in a PicoPure RNA Isolation Kit (Arcturus, CA, USA) and stored at –80°C until use. Total RNAs were prepared from 15 egg cells or 150 sperm cells using the PicoPure RNA Isolation Kit according to the manufacturer’s instructions. Roots and shoots were harvested from 7-days old rice seedlings, and total RNAs were isolated from these tissues using RNeasy Plant Mini Kit (Qiagen, Hilden, Germany). cDNAs were synthesized from total RNAs of egg cells, sperm cells, roots and shoots using the High Capacity RNA-to-cDNA™ Kit (Life Technologies, CA, USA) according to the manufacturer’s instructions. For quantitative PCR analysis, 0.5 µl of first-strand cDNA was used with LightCycler 480 SYBR Green I Master (Roche Applied Science, Penzberg, Germany) according to the manufacturer’s protocol. PCR cycle was conducted as follows: 94°C for 10 s, 55°C for 10 s, and 72°C for 10 s, and relative quantification was calculated with ubiquitin (Os02g0161900) as a reference by the delta delta Ct method. Primer sequences used for PCR analyses are listed in Table S1.

### Fertility of Tos17 Insertional Rice Mutants and T-DNA Insertional Arabidopsis Mutants

Confirmation of the Tos17 insertion at the gene locus was conducted by genomic PCR according to the instructions of the Tos17 database (http://tos.nias.affrc.go.jp/index.html.en) using a left border primer (5′-ATTGTTAGGTTGCAAGTTAGTTAAGA-3′) together with primers specific for the Tos17 insertion lines (Table S2). The mutants with Tos17 mutations were grown in experimental field as mentioned above, and fertility was checked by counting the numbers of developed and undeveloped seeds after harvesting the fully grown plants.

Confirmation of homozygous and heterozygous mutations in *Arabidopsis* mutants was conducted by genomic PCR according to the instructions of the Salk Institute Genomic Analysis Laboratory (http://signal.salk.edu/) using left border primers (LBb3.1, 5′-ATTTTGCCGATTTCGGAAC-3′ for SALK lines; LB3, 5′-TAGCATCTGAATTTCATAACCAATCTCGATACAC-3′ for SAIL lines) with primers specific for the T-DNA insertion lines (Table S3). Seed-set and/or -development were checked by dissecting developing siliques and observing seeds within them.

## Results and Discussion

### SDS-PAGE of Lysates from Isolated Gametes, Somatic Tissues, and Pollen Grains

**Figure 1:**
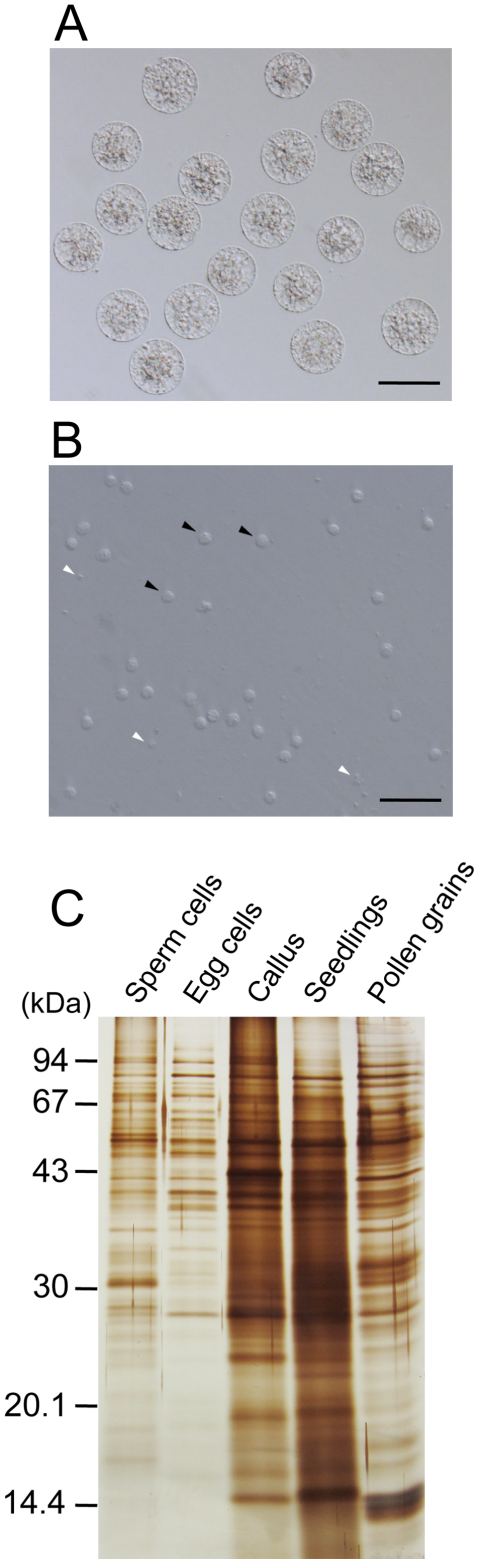
Isolated rice gametes and comparison of protein extracts from rice egg cells, sperm cells, callus, seedlings, and pollen grains. (A) Isolated egg cells. (B) Isolated sperm cells fraction. Black and white arrowheads indicate typical sperm cells and cell debris, respectively. (C) Lysates from 50 egg cells, 3,000 sperm cells, callus (0.1 µg protein), seedlings (0.12 µg protein), and pollen grains (0.06 µg protein) were separated by SDS-PAGE and conventionally silver stained. Bars = 50 µm.

Homogenous egg cells were manually isolated from rice flowers (Figs. 1A and S1). Observation of the sperm cell fraction revealed that sperm cells were almost pure, but cell or tissue debris possibly derived from pollen grains slightly co-existed in the fraction (Fig. 1B).

We first separated lysates from a range (375–6,000) of sperm cells with lysate of 25 egg cells by SDS-PAGE (Fig. S2A). Based on band intensities, we judged that 25 egg cells yielded approximately the same amount of protein as 1,500 sperm cells. Similar calibrations were performed using lysates of callus, seedlings, and pollen grains. Protein yield from 25 egg cells was roughly equivalent to 0.038–0.077 µg callus protein, 0.031–0.062 µg seedling protein (Fig. S2B), and 0.014–0.028 µg pollen-grain protein (data not shown). Based on these comparisons, lysates from 50 egg cells, 3,000 sperm cells, callus (0.1 µg protein), seedlings (0.12 µg protein), and pollen grains (0.06 µg protein) were separated on the same SDS-PAGE gel (Fig. 1C). Protein band intensities for egg and sperm cells were equivalent and approximately 2-3-fold weaker than those of callus, seedlings, and pollen grains (Fig. 1C and S2B). Although protein amounts from callus, seedlings, and pollen grains are higher than those of gametes, in this study, proteome data from somatic tissues and pollen grains were mainly used as subtractive factors to identify gamete-enriched proteins; proteins detected in these tissues were not considered as candidates for gamete-enriched proteins. Therefore, protein amounts of these tissues that were 2-3-fold higher than those of gametes were considered suitable for our purposes. Maintaining this ratio, lysates of 500 egg cells, approximate 3×10^4^ sperm cells, 1 µg of callus proteins, 1.2 µg of seedling proteins, and 0.6 µg of pollen proteins were separated by SDS-PAGE for subsequent LC-MS/MS analyses.

### Identification of Proteins Preferentially Expressed in Rice Gametes

By analyzing proteins from egg and sperm cell lysates, 2,138 and 2,179 proteins were detected, respectively (Tables S4 and S5). Among these proteins, 1,276 and 1,076 proteins were assigned by at least two peptides (Tables S4 and S5). In callus, seedlings, and pollen grains, 2,877, 2,473, and 2,246 proteins were detected, respectively (Tables S6–S8). All proteins from egg cells with >25 identified spectra are listed in Table 1, along with the numbers of identified spectra in the other cell types. Among these proteins, polyubiqutin; molecular chaperones, including heat shock proteins (HSPs) and protein disulfide isomerase (PDI); enzymes of the glycolytic pathway, including phosphoglycerate kinase, glyceraldehyde-3-phosphate dehydrogenase, fructose-bisphosphate aldolase and enolase; ascorbate peroxidase; ATP synthase subunit; and annexin protein were abundantly found. Interestingly, HSP, phosphoglycerate kinase, glyceraldehyde-3-phosphate dehydrogenase, ascorbate peroxidase, and annexin protein were reported as major protein components of rice or maize egg cells in our previous studies [20], [21]. In addition, these housekeeping proteins were also detected in other cell types with at least five identified spectra, suggesting that they can be treated as internal controls between cell types and that finding proteins preferentially expressed in gametes is possible by the use of the number of identified spectra. Next, the number of identified spectra for a protein was compared between cell types to identify the protein preferentially expressing in gametes.

**Table 1:** Proteins with >25 spectra identified in egg cells.

		Number of identified spectra^a^					
cDNA accession	Gene locus	EC	SC	C	S	PG	Protein^b^
AK101547	Os02g0161900	108	48	72	150	96	Polyubiquitin
U37687	Os06g0681400	108	48	72	150	96	Similar to Polyubiquitin protein
AK102389	Os04g0628100	87	39	57	120	77	Similar to Polyubiquitin
AB111810	Os09g0482400	61	18	78	52	36	Heat shock protein 82
AK102426	Os08g0500700	53	15	67	42	28	Similar to Heat shock protein 82
AK061841	Os03g0285700	52	15	56	18	29	Similar to L-ascorbate peroxidase
AK072559	Os06g0221200	47	17	8	19	24	Similar to Annexin p33
AK070041	Os02g0169300	38	24	47	24	47	Similar to Phosphoglycerate kinase, cytosolic
AK065780	Os09g0553200	37	16	45	17	26	Similar to UTP–glucose-1-phosphate uridylyltransferase
AK099086	Os02g0601300	36	21	44	16	39	Similar to Glyceraldehyde-3-phosphate dehydrogenase, cytosolic 3
AK064198	Os03g0260000	36	0	9	0	0	Dynamin family protein
AK061050	Os05g0402700	34	14	33	21	22	Similar to Fructose-bisphosphate aldolase, cytoplasmic isozyme
J100072F13	Os06g0673500	33	15	21	45	29	Similar to Ubiquitin
AK073770	Os01g0905800	32	19	34	18	19	Aldolase C-1
AK068268	Os11g0199200	32	11	31	36	20	Similar to Protein disulfide isomerase
AK064960	Os04g0486600	31	17	33	18	32	Similar to Glyceraldehyde-3-phosphate dehydrogenase, cytosolic 3
AK065431	Os11g0703900	31	17	34	25	28	Heat shock protein 70
AK119528	Os03g0177400	30	10	25	15	13	EF-1 alpha
AK061681	Os05g0553000	30	56	45	23	65	Similar to ATP synthase subunit beta, mitochondrial
AK067757	Os12g0624000	30	10	37	18	24	Similar to Methionine synthase protein
AK069617	Os10g0462900	29	18	45	15	24	Similar to mitochondrial chaperonin-60
AK064953	Os01g0685800	28	55	44	22	64	Similar to ATP synthase beta chain, mitochondrial precursor
AK073999	Os03g0143400	27	13	37	13	22	Similar to mitochondrial chaperonin-60
AK102784	Os03g0821100	26	16	26	14	25	Similar to Non-cell-autonomous heat shock cognate protein 70
AK065255	Os12g0623900	26	9	35	22	21	Similar to Ethylene-responsive methionine synthasesynthase protein
AK073611	Os03g0248600	25	28	44	18	40	Similar to Enolase 2

The number of identified spectra in the egg cell and other cell types are presented. EC, egg cell; SC, sperm cell; C, callus, S, seedling; PG, pollen grain.

a The highest number of spectra assigned for the protein among gel pieces from SDS-PAGE gel of the each cell-type was presented.

b Protein annotations are referred from The Rice Annotation Project Database (RAPDB).

To screen for proteins enriched in egg cells, proteins with >2 identified spectra in egg cells and none in other cell types were selected. In addition, proteins with >3 identified spectra in egg cells and one in another cell type were also chosen as candidates for egg cell-enriched proteins. In total, 109 putative egg-enriched proteins were identified (Table S9). Similarly, 79 proteins were identified as proteins enriched in sperm cells (Table S10). Although pollen-derived cell or tissue debris slightly co-existed in sperm cell fraction (Fig. 1B), subtraction of proteins detected in pollen grains from the list of proteins detected in sperm cell fraction could eliminate the possibility of identifying the pollen-derived proteins as sperm proteins. Table 2 presents putative gamete-enriched proteins with >5 identified spectra. Interestingly, none of these proteins, except for HSP 70 (HSP70), has been reported to play a role in reproductive/developmental processes to our knowledge. Investigating these proteins further may uncover novel molecular mechanisms during gametic development and fusion and early embryogenesis. An abundance of molecular chaperones, including HSPs and PDI, has been suggested to be a common characteristic of mammalian and plant eggs [32]–[34]. In addition, HSPs are suggested to buffer the expression of genetic variation when divergent ecotypes are crossed and profoundly affect developmental plasticity in response to environmental cues [35]. Chaperones in egg cells may function following fertilization by a sperm cell, because conversion of an egg cell into a zygote represents a major genetic and environmental change. Thus, the specific expression of HSP70 in egg cells may be related to fertilization.

**Table 2:** Proteins preferentially expressed in egg or sperm cells with >5 identified spectra.

		Number of identified spectra^a^					
cDNA accession	Gene locus	EC	SC	C	S	PG	Protein^b^
AK106474	Os06g0602400	17	0	0	0	1	Similar to DEAD-box protein 3 (DEAD-box RNA helicase DEAD3)
AK065887	Os03g0283100	15	0	1	0	0	Similar to In2-1 protein
Os06t0706700-01	Os06g0706700	14	0	0	0	0	Similar to PsAD1
AK101183	Os05g0168800	11	0	1	0	0	KIP1-like domain containing protein
AK063589	Os05g0115600	10	0	0	0	0	Protein of unknown function DUF674 family protein
AK106371	Os03g0276800	9	0	0	0	0	Heat shock protein Hsp70 family protein
AK107844	Os05g0143600	9	0	0	0	0	Similar to Jasmonate-induced protein
AK067215	Os01g0698000	8	0	0	0	0	Conserved hypothetical protein
AK063560	Os12g0600100	8	0	0	0	0	Tetratricopeptide-like helical domain containing protein
AK121612	Os02g0717400	8	0	0	0	1	Tetratricopeptide-like helical domain containing protein
AK073477	Os01g0369200	7	0	0	0	0	Similar to Cullin-1
AK058611	Os01g0895100	7	0	0	0	0	Similar to Membrane-associated 30 kDa protein, chloroplast precursor
AK106478	Os01g0771100	7	0	1	0	0	Mitochondrial glycoprotein family protein
AK072587	Os05g0164900	6	0	0	0	0	Galactose oxidase/kelch, beta-propeller domain containing protein
AK067210	Os04g0504800	6	0	1	0	0	Similar to Poly(A)-binding protein
AK072334	Os03g0583900	5	0	0	0	0	DEAD-like helicase, N-terminal domain containing protein
AK119521	Os06g0175800	5	0	0	0	0	Similar to Cystathionine beta-lyase, chloroplast precursor
AK069281	Os09g0471100	5	0	0	0	0	Similar to Peroxidase 17 precursor
AK072719	Os10g0574800	5	0	0	0	0	Similar to ARF GAP-like zinc finger-containing protein ZIGA2
AK064995	Os12g0197500	5	0	0	0	0	Putative Zinc finger, XS and XH domain containing protein
Os01t0876900-00	Os01g0876900	5	0	1	0	0	Conserved hypothetical protein
AK071495	Os11g0255300	0	10	0	0	0	Cysteine endopeptidase
Os01t0267600-01	Os01g0267600	1	10	0	0	0	Sad1/UNC-like, C-terminal domain containing protein
AK065231	Os01g0323100	0	7	0	0	0	Similar to Pto kinase interactor 1
AK099178	Os02g0726000	0	7	0	0	0	FAS1 domain domain containing protein
AK071561	Os05g0163700	0	7	0	0	0	Similar to Acyl-coenzyme A oxidase 4, peroxisomal
AK107034	Os02g0185200	0	6	0	0	0	Cytochrome P450 family protein
AK066587	Os03g0220100	0	6	0	0	0	Similar to Very-long-chain fatty acid condensing enzyme CUT1
Os04g0611200-00	Os04g0611200	0	6	0	1	0	Similar to OSIGBa0152L12.11 protein
AK065311	Os06g0174400	0	6	0	1	0	Similar to Vesicle-associated membrane protein 712
AK069025	Os04g0569000	1	6	0	0	0	Similar to Activator 1 40 kDa subunit (Replication factor C 40 kDa subunit)
AK105867	Os02g0608900	0	5	0	0	0	Similar to Epstein-Barr virus U2-IR2 domain encoding nuclear protein
AK071196	Os05g0399700	0	5	0	0	0	Chitinase
AB087745	Os05g0595100	0	5	1	0	0	Similar to UDP-glucose-4-epimerase
AK069984	Os02g0775200	1	5	0	0	0	Similar to Activator 1 36 kDa subunit (Replication factor C 36 kDa subunit)

The number of identified spectra in all cell types are presented. EC, egg cell; SC, sperm cell; C, callus, S, seedling; PG, pollen grain.

a The highest number of spectra assigned for the protein among gel pieces from SDS-PAGE gel of the each cell-type was presented.

b Protein annotations are referred from The Rice Annotation Project Database (RAPDB).

**Figure 2:**
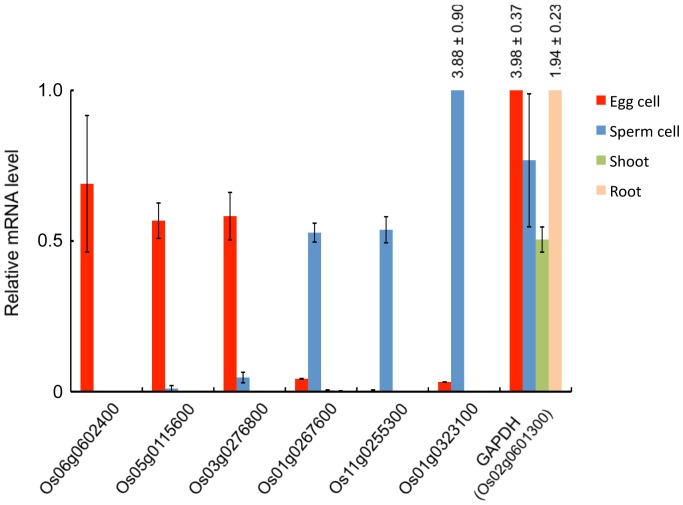
Transcript levels of genes encoding putative gamete-enriched proteins. Transcript levels of six genes encoding putative gamete-enriched proteins and of glyceraldehyde 3-phosphate dehydrogenase (GAPDH) in somatic tissues and gametes were monitored by quantitative PCR and normalized to ubiquitin mRNA. The data are means ±SE of two independent experiments. The numbers at the bars indicate values of relative mRNA levels. See Table S1 for primer sequences.

For monitoring the amount of transcripts for putative gamete-enriched proteins, expression level of six genes listed in Table 2 and a control gene encoding glyceraldehyde 3-phosphate dehydrogenase in somatic tissues and gametes were measured using quantitative RT-PCR. All six genes showed gamete preferential expression (Fig. 2), suggesting that expression levels of gamete-enriched proteins encoded by these genes are regulated at the transcriptional level.

### Fertility of Rice and Arabidopsis Mutants for Putative Genes Encoding Proteins Preferentially Expressed in Egg or Sperm Cells

**Figure 3:**
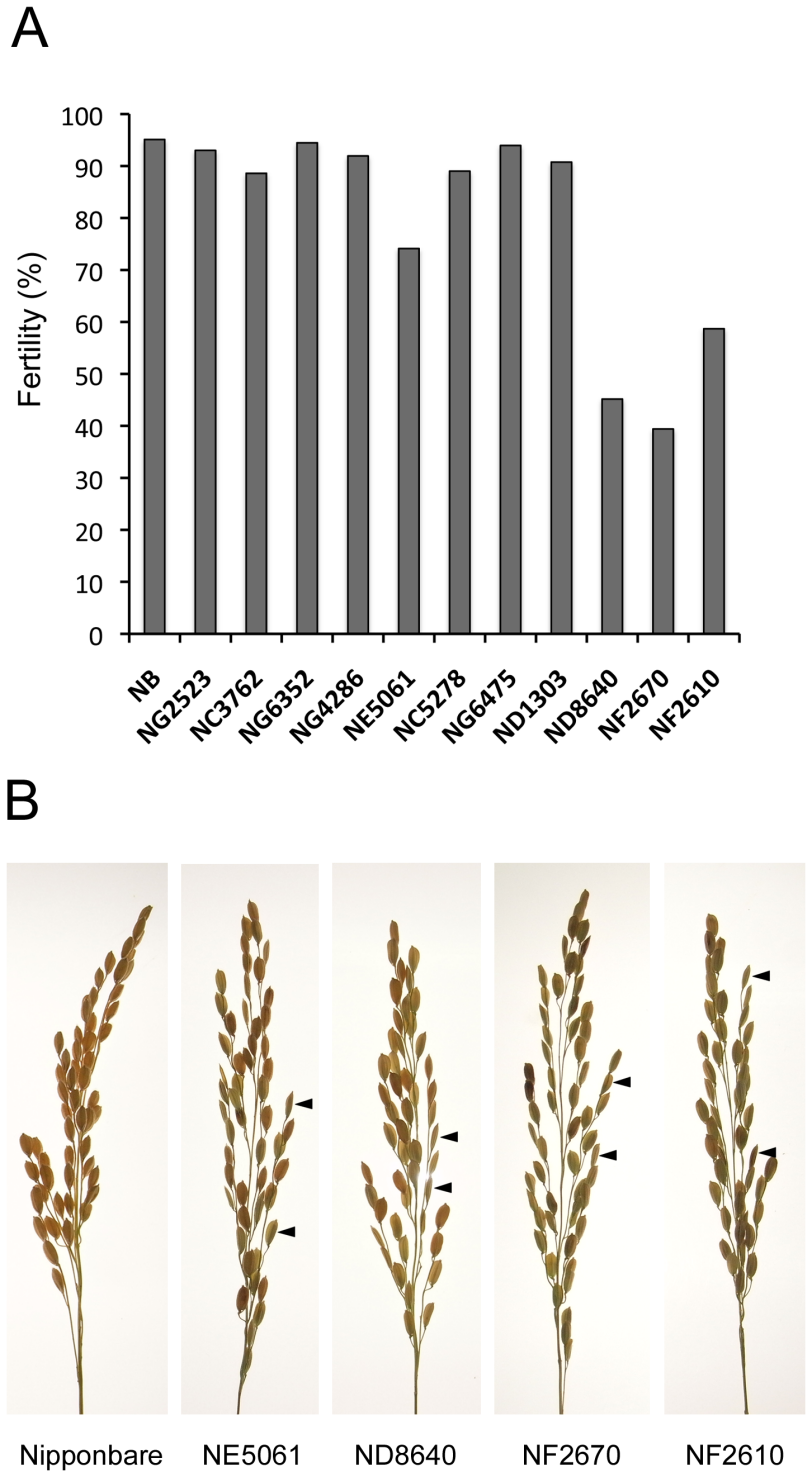
Fertility of rice Tos17 mutants. (A) Percentage seed set of 11 Tos17 mutants. NB indicate wild-type Nipponbare plants. (B) Panicles of Tos17 mutants showing fertility phenotypes. In panicles of Nipponbare, more than 95% seeds developed fully with light brown color. In the four Tos17 mutants, undeveloped seeds were often observed. Two typical undeveloped seeds are indicated by arrowheads in each panel.

Seed-set fertility of plants with mutations in genes encoding gamete-enriched proteins was checked to detect whether functional defects in these proteins could affect reproductive and/or developmental processes such as gamete differentiation, gamete fusion, or early zygotic development. Rice mutants defective in each of the egg cell (109 genes) and sperm cell (79 gene) specific/predominant gene were examined via insertional deactivation of each gene with the Tos17 retrotransposon (Tables S9 and S10) using the Tos17 mutant panel (http://tos.nias.affrc.go.jp/index.html.en). Seven and four Tos17 mutant lines were obtained for egg- and sperm-cell proteins, respectively (Table 3), and their seed-set fertilities were checked. Among these 11 lines, four showed reduced fertility (Fig. 3A and B). Interestingly, three of four mutant lines for sperm proteins showed clear defects in fertility, although only one of seven mutant lines for egg proteins exhibited reduced fertility (Table 3).

**Table 3:** Tos17 insertional rice mutants used to check seed-set fertility.

				Number of identified spectra^a^					
cDNA accession	Gene locus	TOS17 mutant ID	Mutant phenotype	EC	SC	C	S	PG	Protein^b^
AK101183	Os05g0168800	NG2523	no	11	0	1	0	0	KIP1-like domain containing protein
AK063560	Os12g0600100	NC3762	no	8	0	0	0	0	Tetratricopeptide-like helical domain containing protein
AK119521	Os06g0175800	NG6532	no	5	0	0	0	0	Similar to Cystathionine beta-lyase, chloroplast precursor
AK070485	Os05g0555600	NG4286	no	4	0	0	0	1	Similar to NADH dependent Glutamate Synthase precursor
AK067207	Os07g0639600	NE5061	yes	3	0	0	0	0	Peptidase C15, pyroglutamyl peptidase I family protein
AK064563	Os05g0144900	NC5278	no	3	0	1	0	0	Similar to Sucrose-phosphatase
AK064628	Os07g0625500	NG6475	no	3	0	1	0	0	Similar to Fimbriata-associated protein
AK065231	Os01g0323100	ND1303	no	0	7	0	0	0	Similar to Pto kinase interactor 1
Os11t0143400-00	Os11g0143400	ND8640	yes	0	2	0	0	0	DNA-directed RNA polymerase, RPB5 subunit domain containing protein
AK071801	Os04g0223500	NF2670	yes	0	2	0	0	0	Dimethylaniline monooxygenase, N-oxide-forming domain containing protein
Os04t0223901-00	Os04g0223901	NF2610	yes	0	2	0	0	0	Dimethylaniline monooxygenase, N-oxide-forming domain containing protein.

The number of identified spectra in all cell types are presented. EC, egg cell; SC, sperm cell; C, callus, S, seedling; PG, pollen grain.

a The highest number of spectra assigned for the protein among gel pieces from SDS-PAGE gel of the each cell-type was presented.

b Protein annotations are referred from The Rice Annotation Project Database (RAPDB).

**Figure 4:**
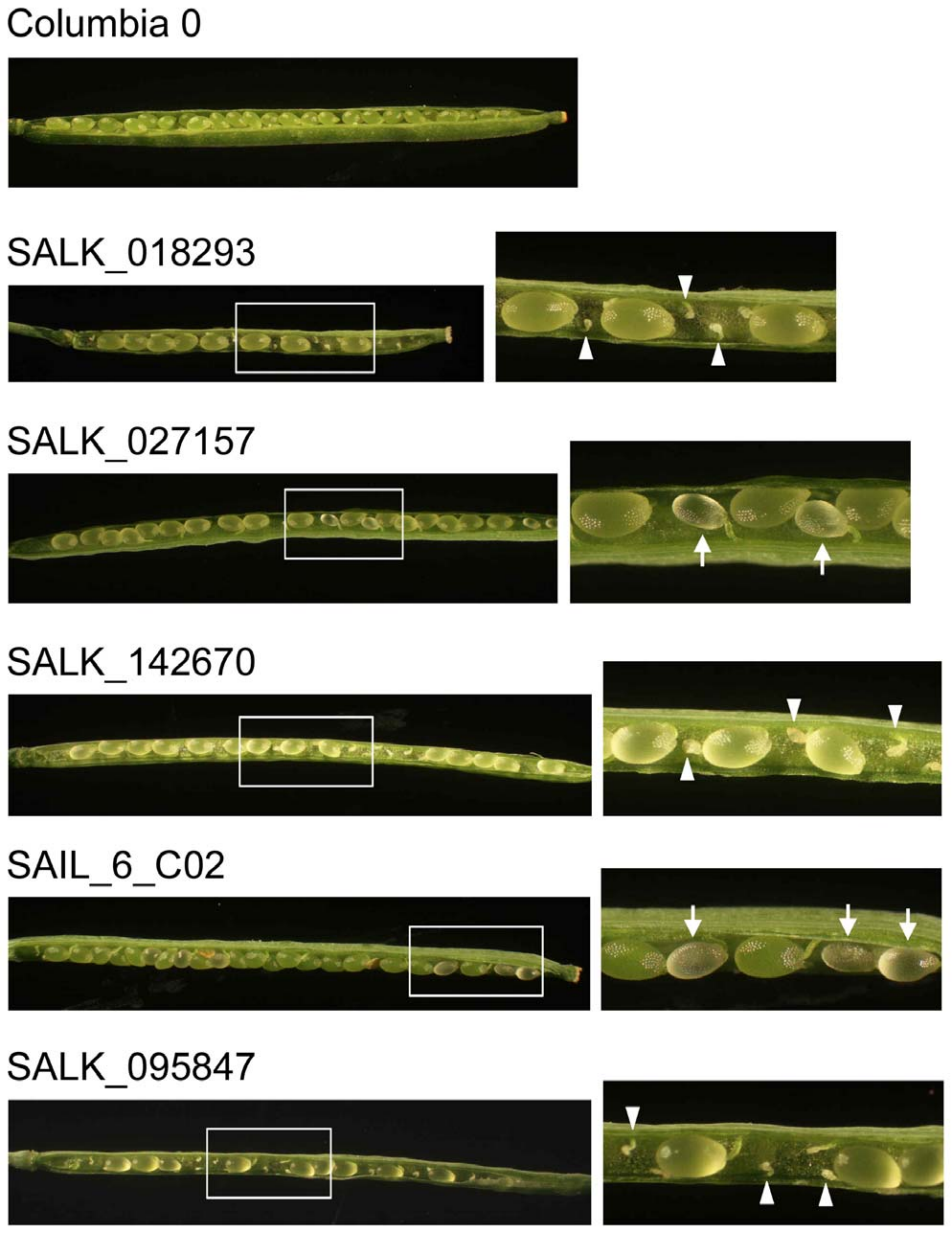
*Arabidopsis* mutants showing defects in seed set or seed development. Panels at right are magnifications of the boxed areas in the left-hand panels. White arrowheads and arrows indicate failed ovules and seeds arrested at immature stages, respectively.

Using rice mutants, we could check the insertional effects of Tos17 on only 11 of 188 genes that were searched using Tos17 mutant panels, probably because Tos17 tends to target to sites within a palindromic consensus sequence, ANGTT-/-AACNT, as cluster [36]. Therefore, we next employed T-DNA insertional mutants of *Arabidopsis*, because abundant mutant stocks are available. First, the *Arabidopsis* genes putatively orthologous to a rice genes encoding gamete-enriched proteins were searched using Surveyed Conserved Motif Alignment Diagram and the Associating Dendrogram (SALAD) database version 1.0 (http://salad.dna.affrc.go.jp/salad/) [37]. *Arabidopsis* orthologs were searched for 61 and 39 genes encoding egg-cell and sperm-cell enriched proteins with >3 identified spectra, respectively (Fig. S3, Tables S8 and S9), resulting in detection of 21 and 13 putative orthologous genes for which 12 and seven T-DNA insertional mutant lines, respectively, were available from ABRC. Seed-set/development in siliques of these mutants was observed. Among these 19 mutant lines, five showed abnormal seed-set phenotypes (Table 4). In developing siliques of three lines (SALK_018293, SALK_142670 and SALK_095847), ovules whose development completely failed were observed (Fig. 4), suggesting that defects in gametophyte formation, gamete function, or fertilization occurred in these mutants. In other lines (SALK_027157 and SAIL_6_C02), seeds arrested at immature stages were observed (Fig. 4), indicating that these mutants may have defects in embryo or endosperm development. We further conducted reciprocal crossing experiments using two mutant lines, SALK_018293 and SAIL_6_C02, since they showed different seed-set phenotypes. In the heterozygous SALK_018293 line, seed fertility was clearly decreased when the mutant pistils were pollinated with wild-type pollen grains or self-pollinated (Table S11). The result suggests that functional defect occurred in female side of the mutant, and may be consistent with that the gene (At3g01910), in which T-DNA is inserted, is orthologous to a rice gene encoding a putative egg-enriched protein (Table 4). However, fertility of the crossed or self-pollinated siliques using heterozygous SALK_018293 line was reduced to one-quarters and the reduction rate appeared to fall too much, since fertility is typically reduced to half when defects in female gametophyte of heterozygous mutant occur. The possibility that the phenotype of SALK_018293 is due to indirect or pleiotropic effects cannot be excluded. When heterozygous SAIL_6_C02 line was used for reciprocal crossing with wild-type, no seed abortion was observed. However, in case of self-pollination of the mutant, seed fertility was reduced to approximately three-quarters, suggesting that homozygous mutation results in defects of post-fertilization events, including embryogenesis and/or endosperm development.

**Table 4:** T-DNA insertional *Arabidopsis* mutants used to observe seed-set and seed-development.

					Number of identified spectra^a^					
cDNA accession	Gene locus	Gene locus of Arabidopsis orthologue	Mutant ID	Mutant phenotype	EC	SC	C	S	PG	Protein^b^
AK058611	Os01g0895100	At1g65260	SAIL_798_C12	no	7	0	0	0	0	Similar to Membrane-associated 30 kDa protein, chloroplast precursor
AK106478	Os01g0771100	At4g32605	SAIL_386_B05	no	7	0	1	0	0	Mitochondrial glycoprotein family protein
AK072587	Os05g0164900	At5g01660	SALK_015342	no	6	0	0	0	0	Galactose oxidase/kelch, beta-propeller domain containing protein
AK072334	Os03g0583900	At1g01040	SALK_081595	no	5	0	0	0	0	DEAD-like helicase, N-terminal domain containing protein
AK119521	Os06g0175800	At3g57050	SALK_034725	no	5	0	0	0	0	Similar to Cystathionine beta-lyase, chloroplast precursor
AK069281	Os09g0471100	At2g22420	SAIL_827_A08	no	5	0	0	0	0	Similar to Peroxidase 17 precursor
Os01t0876900-00	Os01g0876900	At1g71310	SAIL_25_H08	no	5	0	1	0	0	Conserved hypothetical protein
AK068532	Os08g0530400	At3g01910	SALK_018293	yes	4	0	0	1	0	Moco containing protein (OsMCP)
AB049822	Os02g0510200	At3g48560	SAIL_910_E06	no	3	0	0	0	0	Similar to Acetolactate synthase 1, chloroplastic
AK105696	Os02g0754500	At5g09420	SALK_078997	no	3	0	0	0	0	Tetratricopeptide-like helical domain containing protein
AK067167	Os10g0563600	At5g07460	SALK_106118	no	3	0	0	0	0	Similar to Peptide methionine sulfoxide reductase
AK061002	Os01g0276500	At1g31860	SALK_027157	yes	3	0	0	0	1	Similar to Histidine biosynthesis bifunctional protein hisIE, chloroplast
AK121680	Os03g0126000	At5g17990	SALK_142670	yes	3	0	0	0	1	Similar to Phosphorybosyl anthranilate transferase 1
AK069025	Os04g0569000	At1g63160	SAIL_6_C02	yes	1	6	0	0	0	Similar to Activator 1 40 kD subunit (Replication factor C 40 kD subunit)
AK071196	Os05g0399700	At3g12500	SALK_005725	no	0	5	0	0	0	Chitinase
AK073411	Os12g0560700	At4g02060	SALK_095847	yes	0	4	0	0	0	Similar to PROLIFERA protein
AK061335	Os03g0181500	At2g26250	SALK_015616	no	0	4	0	1	0	Similar to Fiddlehead protein
AK072850	Os01g0945800	At4g36960	SALK_068296	no	0	3	0	0	0	Nucleotide-binding, alpha-beta plait domain containing protein
AK104298	Os03g0824400	At1g20570	SALK_051746	no	0	3	0	0	0	Similar to Dolichol-phosphate mannosyltransferase
AK067179	Os05g0401100	At1g54780	SALK_109618	no	0	3	0	1	0	Protein of unknown function DUF477 family protein

The number of identified spectra in all cell types are presented. EC, egg cell; SC, sperm cell; C, callus, S, seedling; PG, pollen grain.

a The highest number of spectra assigned for the protein among gel pieces from SDS-PAGE gel of the each cell-type was presented.

b Protein annotations are referred from The Rice Annotation Project Database (RAPDB).Supporting information Legends

In SAIL_6_C02, function of At1g63160 encoding replication factor C2 (RFC2) is supposed to be defective by T-DNA insertion. Replication factor C is composed of five subunits of RFC1-5, and is known to function in DNA replication, repair and checkpoint control of cell cycles [38]–[40]. Interestingly, Xia et al. (2007) revealed that AtRFC1 plays important role in embryo development [41]. Putative defect of RFC2, a different subunit of RFC, also affected the post-fertilization event, being consistent with the putative function of RFC complex during embryogenesis. For other three Arabidopsis mutants and four TOS17 mutants showing reduced fertility, however, the possible function of the proteins putatively defective in these mutants during reproductive or development is little known. These suggest that further investigations for the molecular functions of these proteins will uncover the novel aspect of plant reproduction and/or development.

### Conclusion

In this study, more than 1,000 proteins expressed in egg cells and sperm cells were globally identified. In addition, we also identified proteins that were preferentially expressed in egg or sperm cells. These data ameliorate the lack of proteomic information for gametes in angiosperms and provide fundamental information for dissecting the specific functions of gametes. Moreover, several rice or *Arabidopsis* lines with mutations in genes encoding putative gamete-enriched proteins clearly showed reproductive or developmental defects. Addressing the functions of these proteins during reproduction and/or zygotic development will improve our knowledge of these processes. Analyses using several mutant plants are currently underway in our laboratories.

## Supporting Information

Figure S1 **Isolation of rice egg cells from ovaries.** (A) A dissected rice flower. (B) Isolated ovary. The dotted line indicates the incision line for egg isolation. (C) Cut ovary. (D) Rice egg cell (arrowhead) being released from basal portion of the dissected ovary.
(TIF)

Figure S2 **Comparison of protein extracts from rice egg cells, sperm cells, callus and seedlings.** (A) Lysates from a range (375–6,000) of sperm cells with lysate of 25 egg cells were separated by SDS-PAGE and conventionally silver stained. (B) Lysates of callus (range: 0.01–0.153 µg protein), seedlings (range: 0.008–0.123 µg protein), and 25 egg cells were separated by SDS-PAGE and conventionally silver stained.
(TIF)

Figure S3 **A typical SALAD analysis result for Os01g0771100 encoding an egg cell-enriched protein.** A dendrogram of sequences clustered according to the presence and similarity of conserved motifs and a diagram that displays positional information of the motifs in each sequence are presented by SALAD database 1.0 [37]. Arrow and arrowhead indicate Os01g0771100 protein and a putative Arabidopsis orthologue. Numbers on dendrogram indicate bootstrapped values, and the same color box presents the same motif.
(TIF)

Table S1 **DNA primers used for quantitative PCR.**
(XLS)

Table S2 **DNA primers used for PCR-based genotyping of rice TOS17 mutants.**
(XLS)

Table S3 **DNA primers used for PCR-based genotyping of** ***Arabidopsis*** **mutants.**
(XLS)

Table S4 **Proteins detected in rice egg cells by LC-MS/MS.**
(XLS)

Table S5 **Proteins detected in rice sperm cells by LC-MS/MS.**
(XLS)

Table S6 **Proteins detected in rice callus by LC-MS/MS.**
(XLS)

Table S7 **Proteins detected in rice seedlings by LC-MS/MS.**
(XLS)

Table S8 **Proteins detected in rice pollen grains by LC-MS/MS.**
(XLS)

Table S9 **Proteins enriched in rice egg cells.**
(XLS)

Table S10 **Proteins enriched in rice sperm cells.**
(XLS)

Table S11 **Reciprocal crossing using SALK_018293 and SAIL_6_C02 mutant lines.**
(XLS)
