# Supplementary figures and images for: Identification of Proteins Enriched in Rice Egg or Sperm Cells by Single-Cell Proteomics
Source: PLoS One. 2013 Jul 25;8(7):e69578. doi: 10.1371/journal.pone.0069578 (PMC3723872; doi:10.1371/journal.pone.0069578)

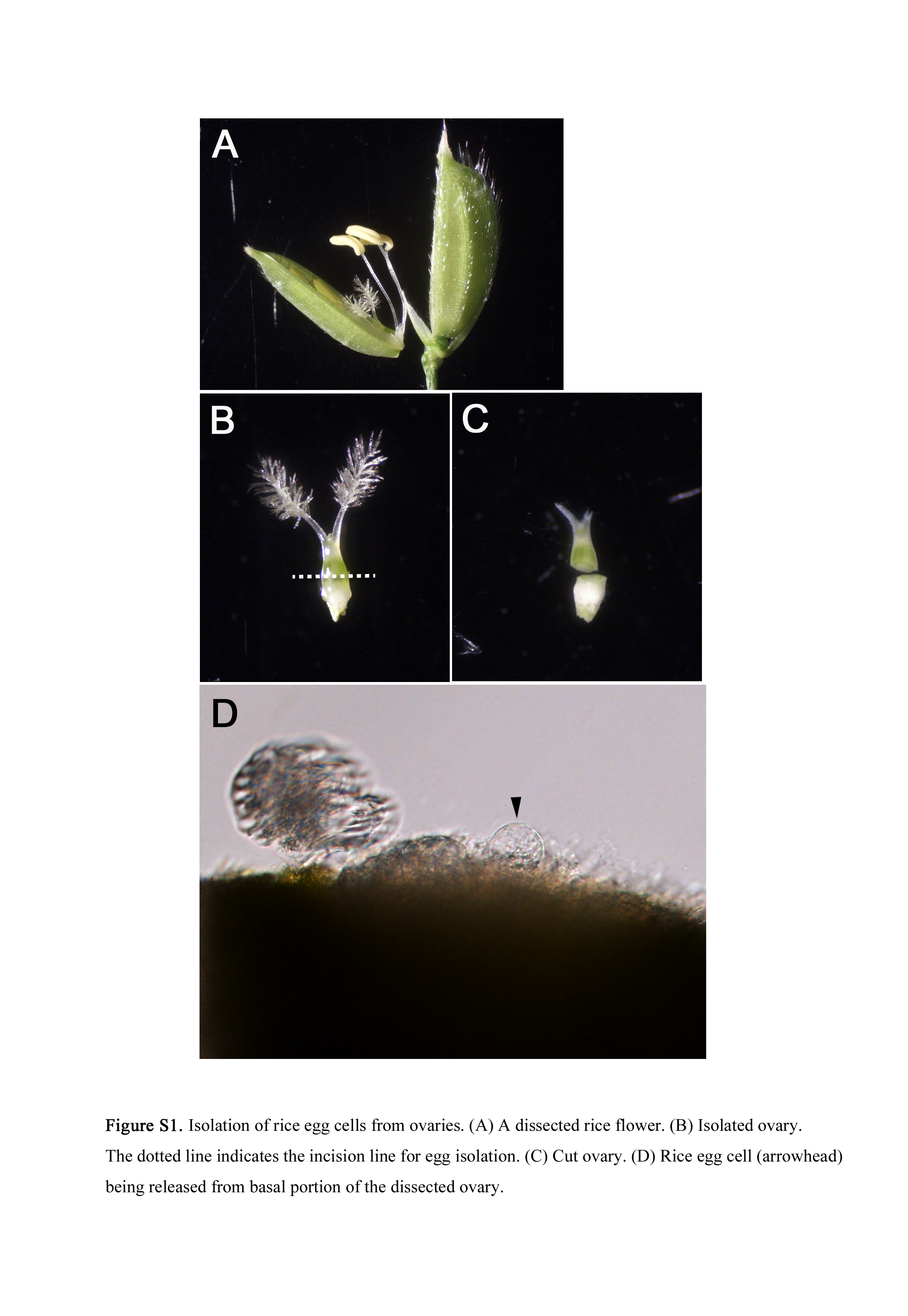

Supplement: Figure S1 — Isolation of rice egg cells from ovaries. (A) A dissected rice flower. (B) Isolated ovary. The dotted line indicates the incision line for egg isolation. (C) Cut ovary. (D) Rice egg cell (arrowhead) being released from basal portion of the dissected ovary. (TIF) [file pone.0069578.s001.tif]

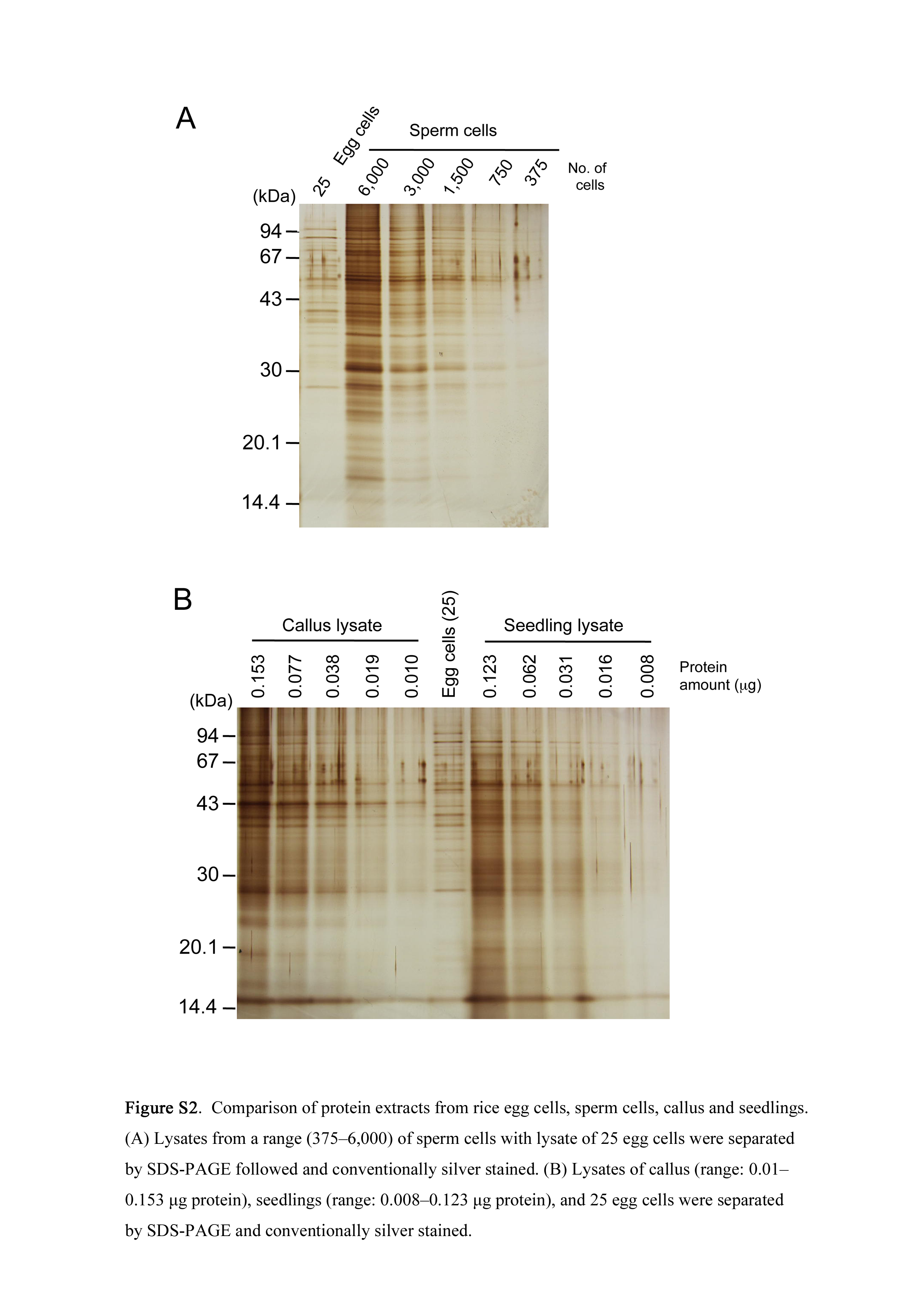

Supplement: Figure S2 — Comparison of protein extracts from rice egg cells, sperm cells, callus and seedlings. (A) Lysates from a range (375–6,000) of sperm cells with lysate of 25 egg cells were separated by SDS-PAGE and conventionally silver stained. (B) Lysates of callus (range: 0.01–0.153 µg protein), seedlings (range: 0.008–0.123 µg protein), and 25 egg cells were separated by SDS-PAGE and conventionally silver stained. (TIF) [file pone.0069578.s002.tif]

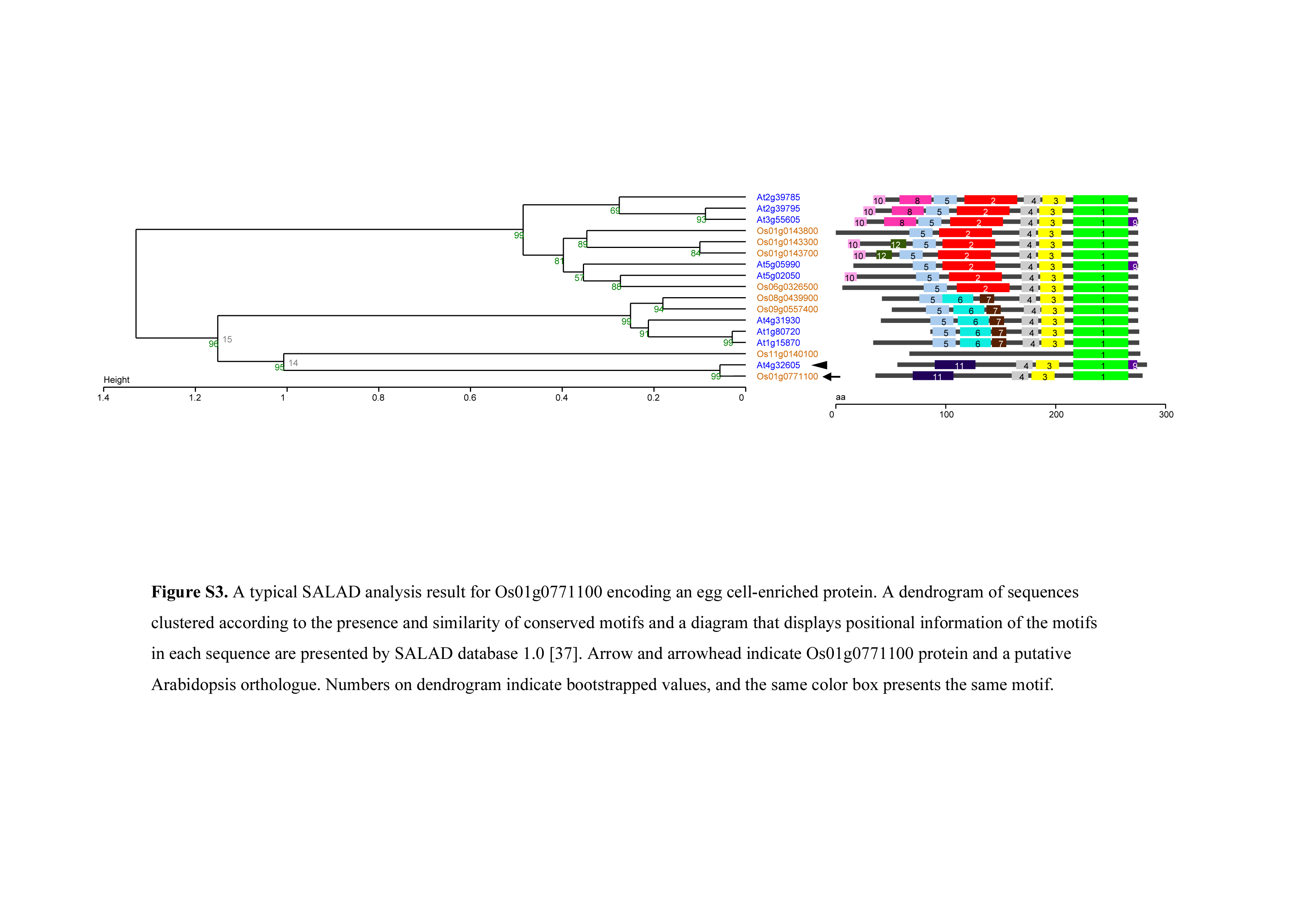

Supplement: Figure S3 — A typical SALAD analysis result for Os01g0771100 encoding an egg cell-enriched protein. A dendrogram of sequences clustered according to the presence and similarity of conserved motifs and a diagram that displays positional information of the motifs in each sequence are presented by SALAD database 1.0 [37]. Arrow and arrowhead indicate Os01g0771100 protein and a putative Arabidopsis orthologue. Numbers on dendrogram indicate bootstrapped values, and the same color box presents the same motif. (TIF) [file pone.0069578.s003.tif]
